# sfkit: a web-based toolkit for secure and federated genomic analysis

**DOI:** 10.1093/nar/gkad464

**Published:** 2023-05-29

**Authors:** Simon Mendelsohn, David Froelicher, Denis Loginov, David Bernick, Bonnie Berger, Hyunghoon Cho

**Affiliations:** Broad Institute of MIT and Harvard, Cambridge, MA, USA; Broad Institute of MIT and Harvard, Cambridge, MA, USA; Computer Science and AI Laboratory, MIT, Cambridge, MA, USA; Broad Institute of MIT and Harvard, Cambridge, MA, USA; Broad Institute of MIT and Harvard, Cambridge, MA, USA; Broad Institute of MIT and Harvard, Cambridge, MA, USA; Computer Science and AI Laboratory, MIT, Cambridge, MA, USA; Department of Mathematics, MIT, Cambridge, MA, USA; Broad Institute of MIT and Harvard, Cambridge, MA, USA

## Abstract

Advances in genomics are increasingly depending upon the ability to analyze large and diverse genomic data collections, which are often difficult to amass due to privacy concerns. Recent works have shown that it is possible to jointly analyze datasets held by multiple parties, while provably preserving the privacy of each party’s dataset using cryptographic techniques. However, these tools have been challenging to use in practice due to the complexities of the required setup and coordination among the parties. We present sfkit, a secure and federated toolkit for collaborative genomic studies, to allow groups of collaborators to easily perform joint analyses of their datasets without compromising privacy. sfkit consists of a web server and a command-line interface, which together support a range of use cases including both auto-configured and user-supplied computational environments. sfkit provides collaborative workflows for the essential tasks of genome-wide association study (GWAS) and principal component analysis (PCA). We envision sfkit becoming a one-stop server for secure collaborative tools for a broad range of genomic analyses. sfkit is open-source and available at: https://sfkit.org.

## Graphical Abstract

**Graphical Abstract:**
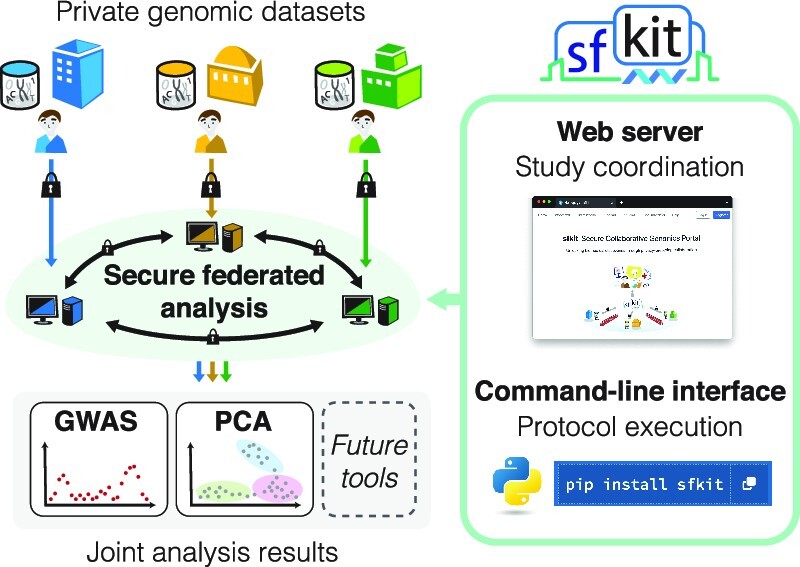

## INTRODUCTION

Data sharing has been a key driving force of progress in genomics. Sharing data across organizations allows researchers to analyze data from larger and more diverse cohorts than what they can individually obtain, which is crucial for extracting novel biomedical insights (1,2). However, biomedical data sharing has become increasingly difficult due to growing concerns about data privacy as well as stricter polices and regulations resulting from these concerns (e.g., the European Union’s General Data Protection Regulation) (3). Creating tools to facilitate the joint analysis of private data across isolated repositories would thus be a great boon for biomedical research.

An emerging field of privacy-preserving data analysis promises tools for jointly analyzing private datasets held by multiple parties while ensuring the privacy of each party’s dataset (3–6). Methods based on cryptographic frameworks for secure computation are especially promising as they provide a formal privacy guarantee that the participating collaborators do not gain information about datasets held by other parties, except the final joint analysis results. Recent works have introduced algorithms built upon these techniques for a range of standard genomic analysis tasks, including genome-wide association studies (GWAS) and principal component analysis (PCA), that can efficiently scale to large datasets including hundreds of thousands of individuals (7–10).

However, leveraging these modern tools for collaboration in real biomedical studies has remained challenging. Applying these methods require a good knowledge of underlying cryptographic techniques, which many biomedical researchers may not immediately possess. Even with such a knowledge, configuring the tools and coordinating interactive computation across a distributed network of computers spanning different organizations would still require substantial time and effort.

To address these challenges, we developed our web server sfkit (Secure and Federated toolKIT for collaborative genomic studies), which streamlines the deployment of secure collaboration tools to broadly enable groups of researchers to easily perform joint analyses of their genomic datasets without the need to share any private data among them. sfkit provides provably-secure collaborative workflows for GWAS and population structure analysis (based on PCA), both built upon state-of-the-art cryptographic techniques (7–10). The design of our web server allows similar methods being developed for a growing range of genomic analysis tasks to be easily incorporated into our server.

In the following, we summarize the system design of sfkit and highlight its key features. We then illustrate the utility and ease-of-use of sfkit for collaborative GWAS and PCA in a range of different settings and datasets. sfkit represents a key step towards broadening researchers’ access to secure collaboration tools for genomics and may help unlock various joint studies that previously could not be realized.

## MATERIALS AND METHODS

### System overview

**Figure 1.**
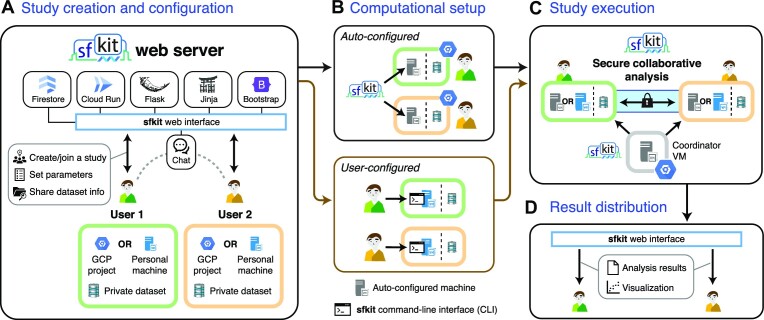
Overview of sfkit workflow. We illustrate the key steps of an example workflow wherein two users securely conduct a joint study using sfkit. (**A**) Using the sfkit web server, the users create a joint study, set analysis parameters, and share (non-private) information about the dataset (e.g., dataset sizes). Chat feature is available to facilitate the communication. (**B**) The users opt to use either their personal machines in *user-configured* mode or automatically instantiated virtual machines (VMs) in their Google Cloud Platform (GCP) projects in *auto-configured* mode. The sfkit command-line interface (CLI) helps streamline the setup in *user-configured* mode. (**C**) sfkit then securely coordinates and executes the study, with minimal user assistance in *user-configured* mode if needed (e.g., to launch the process via the CLI). (**D**) After completion of the study, the users retrieve the joint analysis results and their visualization from the web server.

The sfkit web server provides a common web interface through which users can create, join, configure and run collaborative analyses with other users on a collection of private genomic datasets based on a chosen study workflow (Figure 1). The website implements a range of features, including project bulletin board, chat functions, study parameter configuration and result sharing/visualization, all aimed at streamlining the application of collaborative analysis tools that require complex coordination among multiple users.

To cover a broad range of usage scenarios, sfkit offers two utilization modes: (i) **auto-configured** and (ii) **user-configured**.

In the *auto-configured* mode, the server automatically creates and configures Google Cloud Platform (GCP) virtual machines (VMs) on behalf of users, within the respective user-controlled GCP projects given a minimal set of permissions. This greatly simplifies the setup of networking and computing environments to be used for the joint analysis.

In contrast, the *user-configured* mode allows users who wish to directly configure the machines to provide their own private (or protected) computing environments. To help streamline this latter use case, sfkit additionally provides a user-friendly command-line interface (CLI), which the users can utilize on their machines to communicate with the web server and launch the analysis client program, which in turn communicates with other users’ clients that are executed via the same CLI.

### Security

A key feature of sfkit is the rigorous privacy protection it provides to each user by leveraging state-of-the-art cryptographic tools based on the frameworks of secure multiparty computation (MPC) and homomorphic encryption (HE), both of which enable computation over some form of encrypted information. Throughout the joint analysis workflow, data confidentiality is maintained for each user at all times, except what can be inferred based on the final analysis results. Current workflows operate under the standard semi-honest security model, which assumes that the client programs faithfully follow the protocol as given and aims only to prevent leakage of private information in any intermediate data that is visible to each user during the study execution. Additionally, the workflows employ server-aided preprocessing for computational efficiency, whereby an auxiliary party distributes correlated randomness to the users to accelerate certain cryptographic operations. When needed, sfkit automatically creates a study-specific GCP VM for this role, shown as ‘Coordinator VM’ in Figure 1. Both the web server and the auxiliary VM only facilitate the setup and execution of analysis protocols without receiving any private data from the users. By default, sfkit workflows provide a 128-bit security level, which can be adjusted if desired. All our software modules—the website, CLI, analysis algorithms, and cryptographic libraries (e.g. Lattigo, available at https://github.com/tuneinsight/lattigo)—are open-source, which ensures that our tools are fully transparent. Other methods based on alternative security models (e.g., malicious) are also compatible with our web server. Further technical details of the security of each analysis workflow can be found in the original references (7–10).

### Collaborative analysis workflows

sfkit currently supports the collaborative execution of the following analysis tasks:

#### Genome-wide association study (GWAS)

GWAS is an essential study designed for identifying genetic variants that are correlated with a phenotype of interest, such as disease status or other quantitative biological traits. Analyzing data from a large cohort is crucial for detecting variants that are rare or weakly associated with the trait. The following sfkit workflows allow a group of collaborators to perform an end-to-end GWAS analysis jointly over their datasets, while keeping the input datasets private.

- **MPC-GWAS:** a collaborative GWAS protocol based on secure multiparty computation (MPC) (9). Each user’s input dataset (consisting of genotypes, phenotypes, and covariates for a group of individuals) is split into multiple encrypted copies of the dataset, which are then distributed to collaborators’ machines as input to the joint analysis.
- **SF-GWAS:** a secure and federated (SF) protocol for collaborative GWAS (7,8) based on a hybrid of MPC and multiparty homomorphic encryption (MHE) techniques, which ensures that each input dataset remains locally with the data holder and only smaller amounts of intermediate results are exchanged in an encrypted form.

Both workflows implement a standard GWAS pipeline, including quality control filters (for missing data, allele frequencies, and Hardy-Weinberg equilibrium), population stratification analysis (based on PCA), and association tests using a linear model of the trait based on allele dosages (i.e., Cochran-Armitage test for binary traits). Other workflows based on logistic or linear mixed models will be supported in a subsequent version.

We expect the MPC-GWAS and SF-GWAS workflows to be useful in different settings: MPC-GWAS allows each user to provide only an encrypted version of their dataset as input to the sfkit workflow, simplifying trust assumptions. Although SF-GWAS requires that user’s original input dataset be available on the user’s machine, this allows the sfkit protocol to leverage efficient local computation on the unencrypted data to greatly reduce runtime requirements (7).

#### Principal component analysis (PCA)

PCA is a standard algorithm for dimensionality reduction, commonly applied in genetic studies to identify the population structure of a given cohort. Coordinates of each individual in a reduced space output by PCA are thought to represent their genetic ancestry in relation to other individuals in the dataset. This information is useful in various settings, e.g., for defining study cohorts or constructing additional covariate features in GWAS.

- **SF-PCA:** a secure and federated (SF) protocol for a group of users to perform a PCA jointly on their private datasets to obtain a desired number of top principal components (PCs) (10). This corresponds to one of the steps in the GWAS workflows above, here provided as a standalone tool. Each user provides a matrix with the same number of columns (features) as the local input dataset.

### Usage process

sfkit securely executes collaborative workflows in four main steps outlined below. Note that in the *auto-configured* mode, users only need to create the study and grant sfkit limited access to their GCP project; all subsequent steps are orchestrated and automatically executed by sfkit. In the *user-configured* mode, users run all the steps after study creation and configuration in their own (private) environment using the sfkit CLI. We illustrate the workflow in Figure 1 and showcase sfkit’s user interface in Supplementary Figures S3–S5.

1. **Study creation and configuration**. Users navigate to the ‘Studies’ page of the website to create a study (or join an existing study). When creating a new study, they select or enter configuration options, study name, parameters, and other study details. Once the study has been created, other participants can join the study either through a request button on the ‘Studies’ page (which requires approval by the study creator) or privately via an invitation button. Note that registration and login are optional; users can choose to create or join studies anonymously if they prefer. In this case, a unique permanent link will be provided to them so they can return to the study later. In both utilization modes, sfkit’s web server stores only the study’s information (e.g. name and description) and analysis parameters.
2. **Computational setup**. In the *auto-configured* mode, users are guided through the setup of a GCP project containing their data, ensuring compatibility with sfkit. Users enter information about their GCP project, allowing sfkit to set up the networking and compute resources necessary to run the analysis. In the *user-configured* mode, users provide their own networking and compute environments (e.g. the IP address) and interact with the web server via the sfkit CLI.
3. **Study Execution**. The study is executed in three steps:
  - **Key exchange**. Participants generate cryptographic key pairs, with public keys being exchanged among them via sfkit. It is important to note that the required private keys are generated locally on participants’ machines and never revealed to any other entity, including sfkit’s web server.
  - **Data validation**. Each participant’s data is validated locally on their machine to ensure compliance with the appropriate format for the study and its parameters. No private information is exchanged during this step.
  - **Protocol execution**. The chosen analysis (e.g. GWAS) is performed using the corresponding secure collaborative protocol. Status updates appear on the study page. Users can leave the page and check back periodically for study completion updates.
  In *auto-configured* mode, users click a single button on the website saying ‘Begin Workflow,’ which automatically performs the above steps. In *user-configured* mode, these steps are performed via the CLI.
4. **Distribution and visualization of results**. Upon study completion, results are optionally visualized in sfkit’s web interface, and users can download them. For the GWAS workflows, the results for each user consist of global association statistics. For the PCA workflow, the results for each user comprise a projection of their data onto the top principal components.

### Web server implementation

sfkit is an open-source web server built on Google Cloud Platform (GCP) utilizing Flask (https://flask.palletsprojects.com/), and consists of three primary layers: web server, database, and computational machines. The web server layer, hosted on Google Cloud Run, employs Jinja (https://jinja.palletsprojects.com/), Bootstrap (https://getbootstrap.com/), and custom components for a responsive user interface. The database layer, hosted on Google Cloud Firestore, manages studies, parameters, and website functionalities. The machine layer, on Google Cloud Compute Engine, performs computations using Python-based sfkit CLI, with core protocols implemented in C++ and Golang.

### Command-line interface (CLI) implementation

The CLI, developed in Python, serves as a secure bridge between the user’s compute environment and the web server, facilitating core protocol execution. It uses libraries such as PyNaCl (https://pypi.org/project/PyNaCl/) for encryption; and requests (https://pypi.org/project/requests/) for server communication. Available on PyPI, the CLI features a modular design for easy integration of new protocols. In *user-configured* mode, users follow a guided process using token-based authentication for secure connection. The CLI offers a suite of commands for environment configuration, input validation, and protocol initiation and execution. These commands interact with the sfkit web server (hosted in GCP) for coordination purposes, but do not send any private data to the server. In *auto-configured* mode, the coordinating VM automates CLI commands, streamlining the process while maintaining security and reliability.

### Software documentation and tutorials

Detailed documentation and instructions for using sfkit are publicly available on two online resources: the sfkit website at https://sfkit.org/instructions and the command-line interface documentation at https://sfkit.readthedocs.io/. These resources provide information on how to perform an analysis using sfkit, whether using the *auto-configured* or *user-configured* mode. They also offer guidance on how to prepare the input data and describe every step of the workflow.

## RESULTS

### A case study: consortium-based collaborative analysis

We applied sfkit workflows to analyze a collection of genomic datasets from the eMERGE consortium, which included a total of 31,292 individuals split across seven data collection sites (Supplementary Note 1). To demonstrate a collaborative study, we simulated a user for each site with access to the corresponding data subset. In the following, we describe the results from the perspective of these users.

The users utilized sfkit’s *auto-configured* mode, in which the server automatically created a virtual machine with 16 CPUs and 128 GB RAM on GCP for each user. For the GWAS analysis, the users adopted a common set of 38,040,168 imputed biallelic SNPs and 9 covariate features to include in the analysis, and chose body-mass index (BMI) as the target phenotype. More details about the dataset and the analysis parameters can be found in Supplementary Note 1.

**Figure 2.**
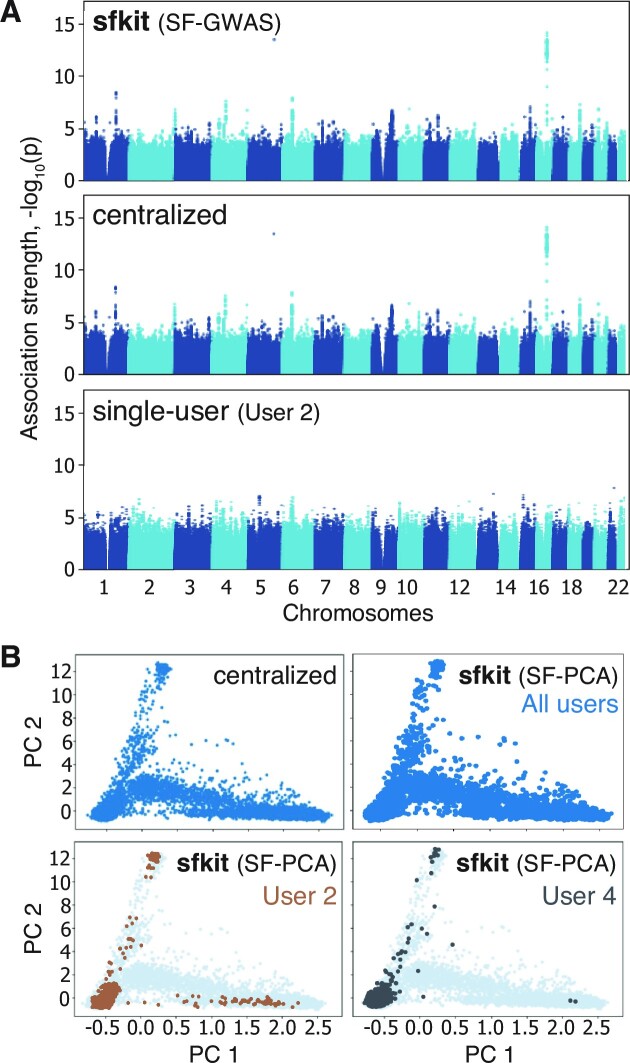
Example collaborative GWAS and PCA analyses using sfkit on the eMERGE dataset. (**A**) We show the Manhattan plot obtained by the collaborators when using sfkit to jointly perform a GWAS on the eMERGE dataset, split among seven data collection sites. With sfkit, the collaborators securely obtain the same results as a centralized study on the pooled dataset, whereas a single user alone does not have sufficient statistical power to detect associations. (**B**) Individuals are projected onto the top two principal components (PCs) obtained by different approaches, with examples shown for users 2 and 4 (see Supplementary Figure S2 for the remaining users). The combined results of all users confirm that with sfkit the collaborators obtain comparable results to a PCA conducted on the pooled dataset.

After the automatic execution of the SF-GWAS workflow using sfkit, each participating user obtained association statistics that are nearly identical to the equivalent study executed on the pooled dataset (Figure 2A). The top two loci with the strongest association signals identified by sfkit were co-located with *SLC25A48* and *FTO* genes, recapitulating previously reported genetic factors of obesity (11,12). In contrast, a single user analyzing their own dataset (comprising 1827 samples) obtained far fewer significant associations (one with *P* < 5 × 10^−8^, compared to 73 for sfkit), which illustrates the increased power of collaborative analysis enabled by sfkit (Figure 2A). Meta-analysis led to substantially different results compared to the pooled analysis in our setting (Supplementary Figure S1).

Similarly for PCA, the users obtained joint analysis results using sfkit that were highly consistent with a centralized study (Figure 2B). The top two PCs revealed the structure of differing ancestry backgrounds among individuals in the cohort. Note that each user obtains the projections of only the individuals in their local dataset onto the top PCs (Figure 2B). sfkit allows the use of PCs that are jointly constructed across the users, which is otherwise not possible if the datasets cannot be pooled. These projections can in turn be used as covariates in GWAS to correct for population stratification.

sfkit greatly simplifies the setup of the joint analysis among seven parties down to a small number of interactions on the website and deploys the computational protocol in less than five minutes. The entire GWAS computation is executed in seventeen hours and PCA in 3 hours (Supplementary Table S1).

### Reproducible tutorial demonstration on a public dataset

**Figure 3.**
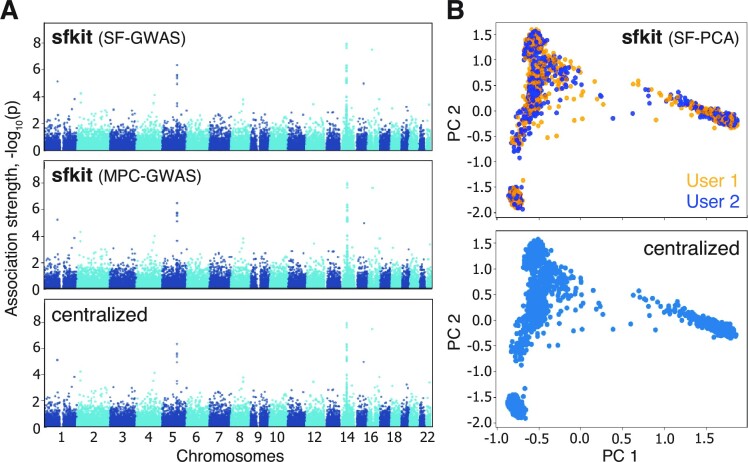
Reproducible demonstration of sfkit workflows on the public 1000 Genomes Project dataset. sfkit allows users to perform both GWAS (**A**) and PCA (**B**) securely on distributed datasets, while achieving results that are comparable to those obtained from centralized studies, executed using PLINK (13) on the corresponding pooled dataset. Two distinct workflows for GWAS are shown (SF-GWAS and MPC-GWAS), which perform similar computation, but provide different security properties (Materials and Methods). These examples can be reproduced by following a tutorial on the sfkit web server.

We additionally demonstrate all three workflows of sfkit on a public dataset (1000 Genomes Project; Supplementary Note 1), which can be reproduced following our step-by-step tutorial on the web server. For GWAS, we simulated both covariate features and phenotypes based on a small set of causal variants. All three workflows resulted in an output that closely agree with that of non-secure centralized studies on the pooled dataset within two hours of runtime for each workflow (Figure 3; Supplementary Table S1).

### Alternative utilization modes and environments

We extensively tested sfkit’s workflows using both utilization modes (*auto-configured* and *user-configured*) and in different computational environments (using machines that are co-located in GCP versus hosted by different cloud providers). For example, we reproduced the experiments from the previous section, but this time between a user using GCP and another user using Azure to host their machines, both utilizing the *user-configured* mode. These variations in settings did not impact the accuracy of sfkit’s workflows and produced identical results. The additional delay introduced by more distant/cross-platform connections remained manageable; e.g., runtime to perform SF-GWAS on the 1000 Genomes Project dataset increased from 110 to 143 min. These experiments are summarized in Supplementary Table S1.

### Runtime and monetary cost

**Figure 4.**
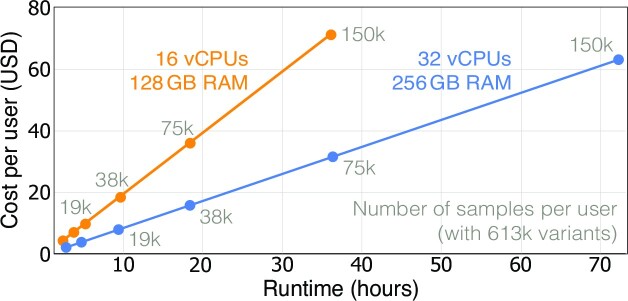
Scaling of cost and runtime for collaborative analyses using sfkit. When using sfkit in the *auto-configured* mode, virtual machines (VMs) are automatically provisioned for users in the Google Cloud Platform (GCP) based on their specified parameters. We illustrate the impact of dataset size and VM type on both runtime and monetary cost of sfkit based on the SF-GWAS workflow. We replicated the Lung Cancer dataset (9178 samples with 613k variants) to obtain datasets of varying sizes, then split each dataset between two users. Runtime and cost remain practical for large datasets, and using a higher-class VM can further reduce the runtime. vCPU: virtual CPU.

We evaluated the cost of sfkit on a range of dataset sizes obtained by replicating the Lung Cancer dataset (Supplementary Note 1). We split each dataset evenly between two users and ran the SF-GWAS workflow. The cost of a study increased linearly with the study’s runtime (Figure 4). sfkit allows users to choose from a range of virtual machine (VM) types to strike a balance between runtime and monetary cost. For instance, by opting for a more powerful machine (with 32 CPUs instead of 16), the runtime on a dataset with 150k samples per user could be reduced by half, albeit at an increased cost of $70 instead of $62 per user (in USD). By extrapolating these results, we estimate a cost of $200 per user even on a much larger dataset including, e.g. 200k samples and 90 milion SNPs. Further cost reductions may be possible with more optimized usage of cloud computing services.

### Related work

To the best of our knowledge, sfkit is the only existing web server that automates the execution of a collection of cryptographic algorithms developed for collaborative genomic analysis workflows with a provably high level of security.

Several strategies and software tools have been developed for collaborative GWAS (13–16). The PLINK software (https://www.cog-genomics.org/plink/) implements *meta-analysis* methods, allowing multiple parties to statistically combine their local GWAS results without sharing individual-level data. Nasirigerdeh et al. (14) proposed sPLINK, a federated implementation of GWAS with secure aggregation of local statistics, which is also available as a web-based tool. Both these approaches do not support a collaborative PCA, an essential step in GWAS. They also require the users to install the required software and setup their own machine, processes that are automated in the *auto-configured* mode of sfkit. Moreover, these existing approaches still reveal some aggregated intermediate results between the participants. It has been shown that the shared intermediate results in federated analysis pipelines can reveal some information about the private input datasets (17,18). With sfkit, no data is revealed except for the final analysis results. We further note that meta-analysis can be less accurate than a centralized study, especially given heterogeneous data distributions (14,19) (Supplementary Figure S1).

Several general-purpose and open-source software have been developed for federated model training and data analysis, including: FedML (https://www.fedml.ai/), FATE (https://fate.fedai.org/), PySyft (https://blog.openmined.org/tag/pysyft/), OpenFL (https://github.com/intel/openfl) and TensorFlow Federated (https://www.tensorflow.org/federated). While some of these solutions provide a similar level of privacy protection as sfkit (e.g. PySyft), none of them are designed for genomic analyses. Adapting these existing tools to efficiently perform the sophisticated workflows addressed by sfkit would require substantial effort.

## CONCLUSION

sfkit is a user-friendly web server designed to help a group of researchers securely perform collaborative genomic analyses, including association tests and population stratification analysis, jointly across datasets that cannot be pooled together. The modular design of sfkit facilitates seamless integration of additional analysis workflows, such as training of disease risk prediction models. A key direction for future work is to demonstrate analysis across large-scale biobanks, e.g., the All of Us Research Program and the UK Biobank, leveraging sfkit’s capabilities. sfkit represents a step toward broadening access to state-of-the-art cryptographic tools for collaborative biomedical research.

## DATA AVAILABILITY

The eMERGE and Lung Cancer datasets are available through the National Institutes of Health’s database of Genotypes and Phenotypes (dbGaP) under accession numbers phs000888.v1.p1 and phs000716.v1.p1. The example dataset constructed based on the 1000 Genomes Project dataset is available for download on the tutorial page of our website.

## Supplementary Material

gkad464_Supplemental_File
