# Supplementary material for: sfkit: a web-based toolkit for secure and federated genomic analysis
Source: Nucleic Acids Res. 2023 May 29;51(W1):W535–41. doi: 10.1093/nar/gkad464 (PMC10320181; doi:10.1093/nar/gkad464)
Supplement: gkad464_Supplemental_File [file gkad464_supplemental_file.pdf]

Supplementary Note 1: Datasets and Experimental Details

Our experiments involved the use of three distinct datasets: eMERGE, 1000 Genomes Project, and Lung Cancer (see Data Availability section). To conduct GWAS and PCA analyses on these datasets, we employed both cryptographic and algorithmic parameters similar to those utilized in prior works introducing the methods supported by our web server (e.g., see MPC-GWAS [1] and SF-GWAS [2]).

eMERGE dataset. The eMERGE dataset we obtained comprises a cohort of 31,292 individuals in total, distributed across seven study groups: Geisinger Health System (containing n=3,089 samples), Group Health (University of Washington; n=1,827), Marshfield Clinic (Pennsylvania State University; n=4,736), Mayo Clinic (n=6,208), Icahn School of Medicine at Mount Sinai (n=5,661), Northwestern University (n=4,424), and Vanderbilt University (n=5,347). We simulated a distinct user for each study group to demonstrate a collaborative study on this dataset. For the GWAS analysis, the users agreed upon a set of 38,040,168 imputed biallelic SNPs and chose body-mass index (BMI) as the target phenotype. They also included the following covariates: membership in each site, age (at the time of assessment), age^2 and sex. For the quality control (QC) step in GWAS, we used the following parameters: genotype missing rate per SNP < 0.1, minor allele frequency (MAF) > 0.05, and Hardy–Weinberg equilibrium chi-squared test statistic < 23.928 (p-value $> 10^{-6}$). Following QC filtering (applied jointly across all users' datasets), we then applied a minimum pairwise distance threshold of 100 Kbp to select a subset of 35,334 uncorrelated SNPs to use in the PCA step, wherein we used five principal components.

1000 Genomes Project dataset. The dataset we obtained from the 1000 Genomes Project contains a subset of 28,021 SNPs over 2,504 individuals (split evenly among two participants). We downsampled the SNPs to allow prospective users to more quickly reproduce our results for tutorial purposes. We chose significant SNPs and then simulated the phenotype data using the GCTA GWAS Simulator [3]. We also randomly sampled two covariates, representing sex and age, independently of the phenotype. We used the following filters for quality control: genotype missing rate per SNP < 0.1, minor allele frequency (MAF) > 0.01, and Hardy–Weinberg equilibrium chi-squared test statistic < 28.374 (p-value $> 10^{-7}$). For PCA step, we used five PCs and imposed a minimum pairwise distance threshold of 100 Kbp after QC filtering between the SNPs to obtain a reduced set of SNPs with low levels of linkage disequilibrium.

Lung Cancer dataset. The lung cancer dataset includes 612,794 autosomal SNPs across a total of 9,178 individuals (5,088 cases and 4,090 controls). In our experiments, we evenly divided the data among two collaborators. The study cohort was divided into five age groups: < 40, 40–50, 50–60, 60–70, and > 70. In accordance with previous works [1, 4], we used binary membership vectors for age and study group as additional covariates for the association tests (10 linearly-independent features) and applied the following filters for quality control: genotype missing rate per individual < 0.05 and per SNP < 0.1, individual heterozygosity rate > 0.25 and < 0.30, minor allele frequency > 0.1, and Hardy–Weinberg equilibrium test chi-squared statistic < 28.3740 (P-value $< 10^{-7}$). We followed the same procedure for population stratification as we did for the eMERGE dataset, which resulted in a total of 23,724 loci being used for PCA (five PCs).

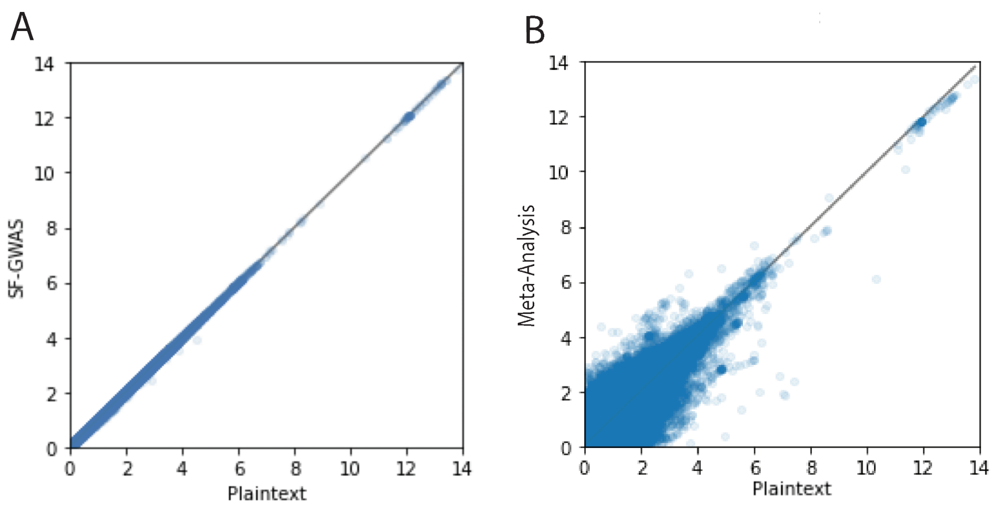

Supplementary Figure 1: **sfkit allows users to obtain accurate joint GWAS results.** In this example, we simulated seven users utilizing sfkit to conduct a GWAS on the federated eMERGE dataset (see Supplementary Note 1). (A) We observe that with sfkit the users obtain results that are highly consistent with those of a centralized study for all SNPs (including those that are non-significant). (B) On the other hand, users combining their local GWAS results through a meta-analysis (using the PLINK software [5] with the fixed-effect model) obtain results that considerably deviate from those of a centralized study in our setting.

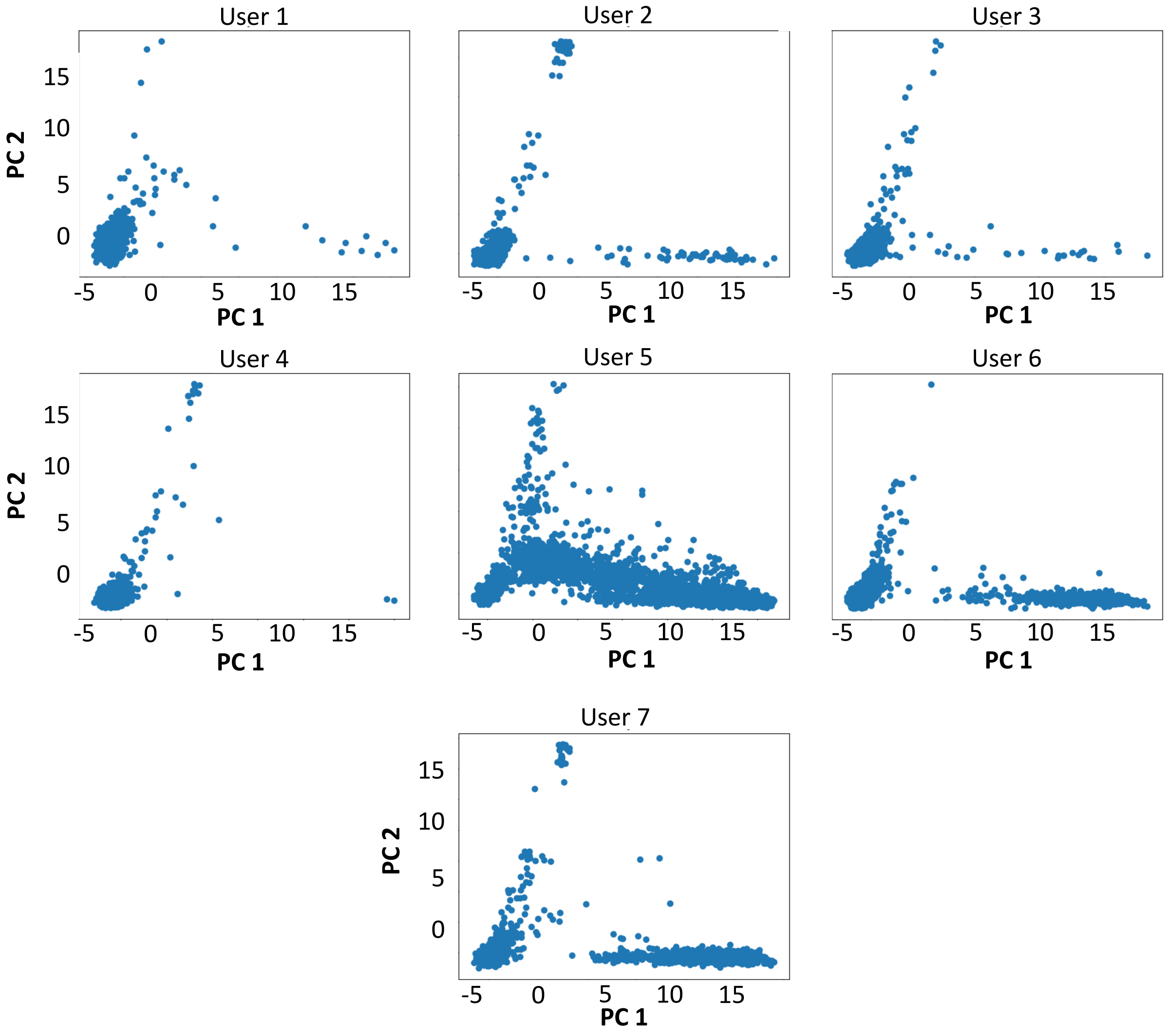

Supplementary Figure 2: Projections on the first two principal components obtained by each user through the use of sfkit. In this example, seven users collaboratively conduct a PCA on the federated eMERGE dataset (see Supplementary Note 1). While the principal components are globally computed by jointly considering all users' datasets, each user obtains only the projection of their own data and no other information about the datasets contributed by other parties.

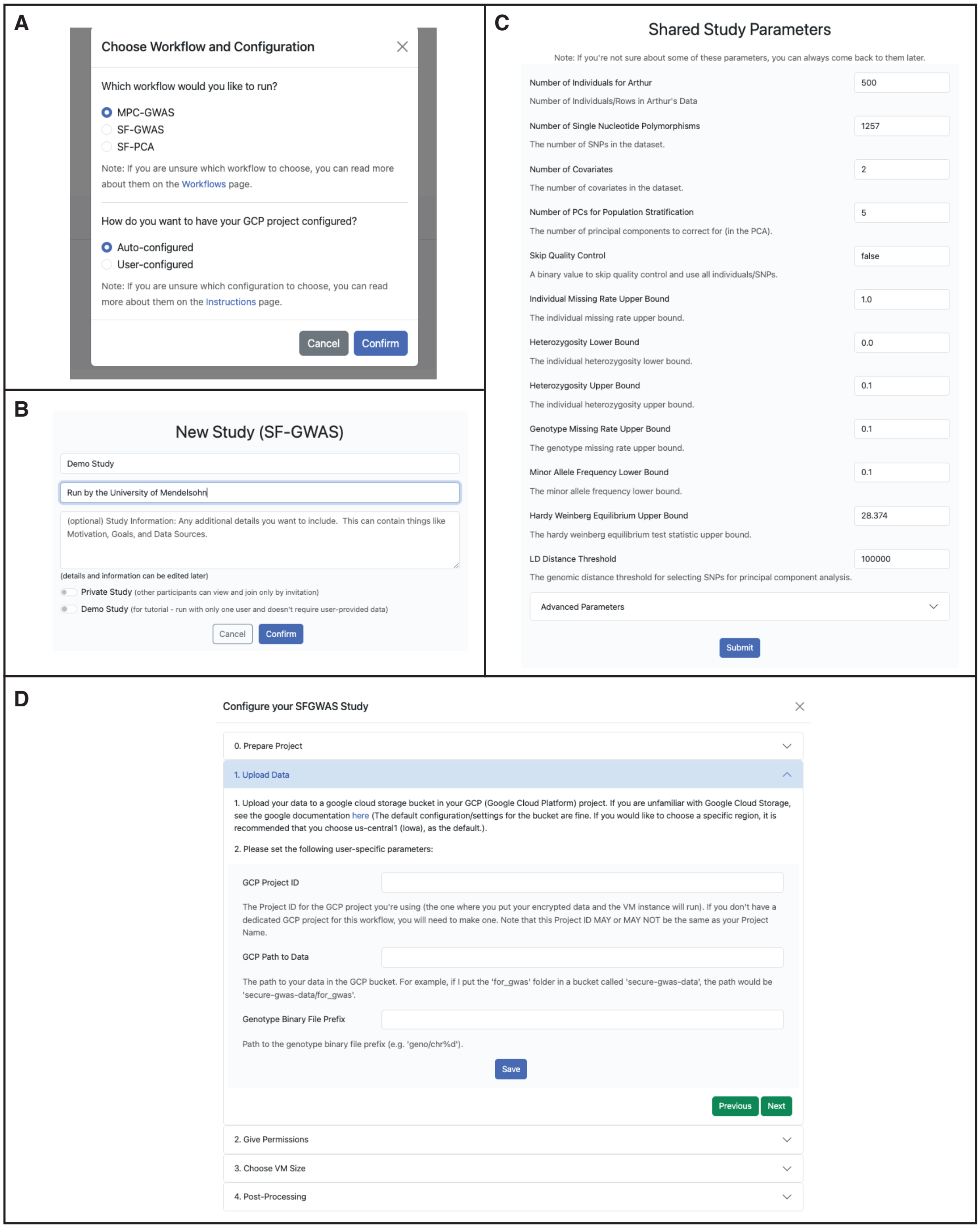

Supplementary Figure 3: **Screenshots of the steps for setting up an *auto-configured* study. A** and **B** show screenshots of the user interface for study creation (encompassing workflow selection, configuration options, and inputting study name, description, and additional information), while **C** displays the interface for editing study parameters, with descriptions and default suggested values for ease of use. In **D**, we showcase one of the steps of the configuration walk-through, wherein the user is prompted to provide information about their GCP Project and data locations.

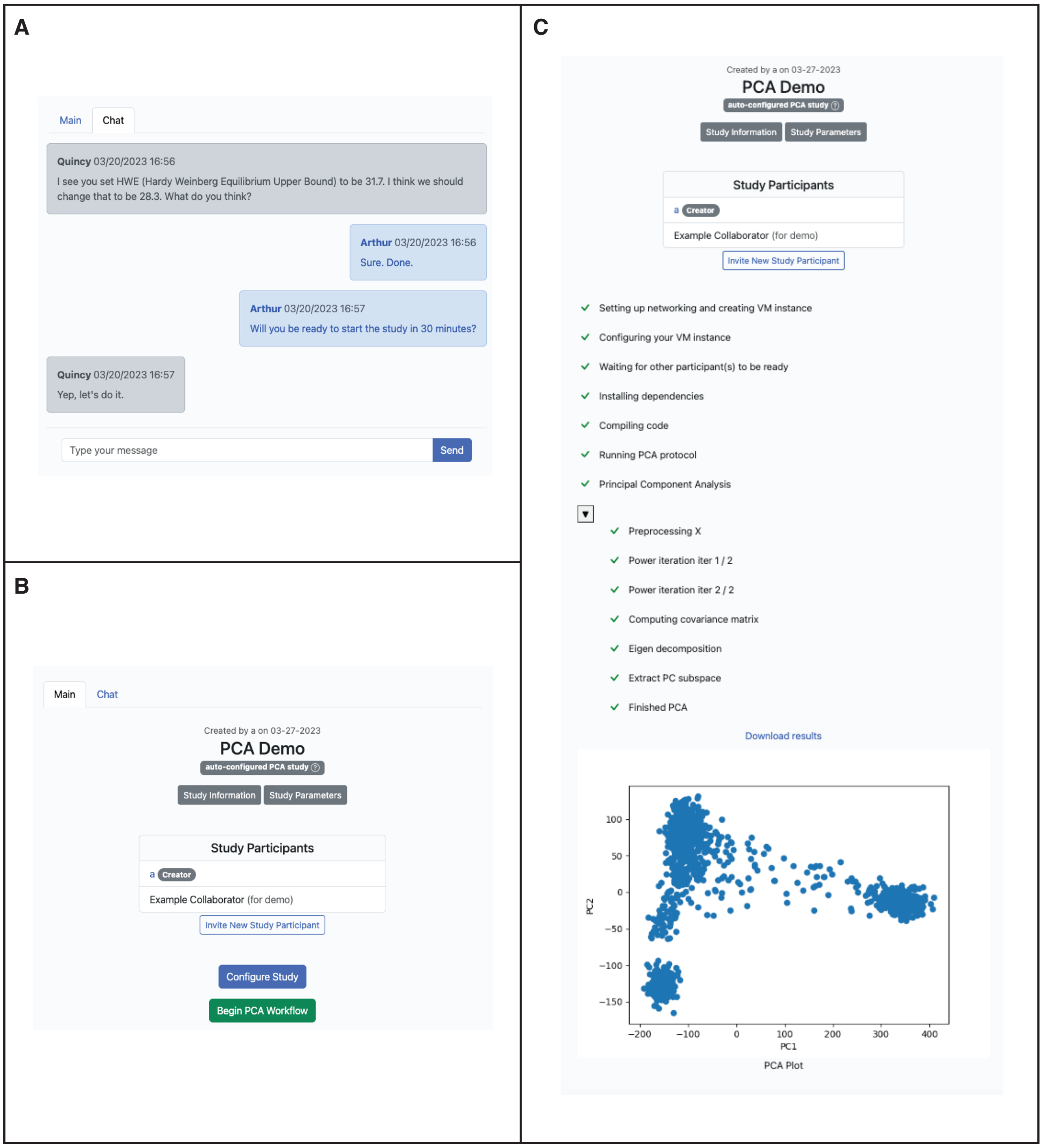

Supplementary Figure 4: **Screenshots of the steps for executing an *auto-configured* study.** As an example use of the chat function, we illustrate participants chatting to confirm readiness (**A**) before initiating the protocol by clicking the "Begin PCA Workflow" button (**B**). (**C**) We show the project page of an ongoing/completed study, wherein live updates on completed steps throughout the execution process can be seen. It also includes a visualization of the final results generated upon study completion.

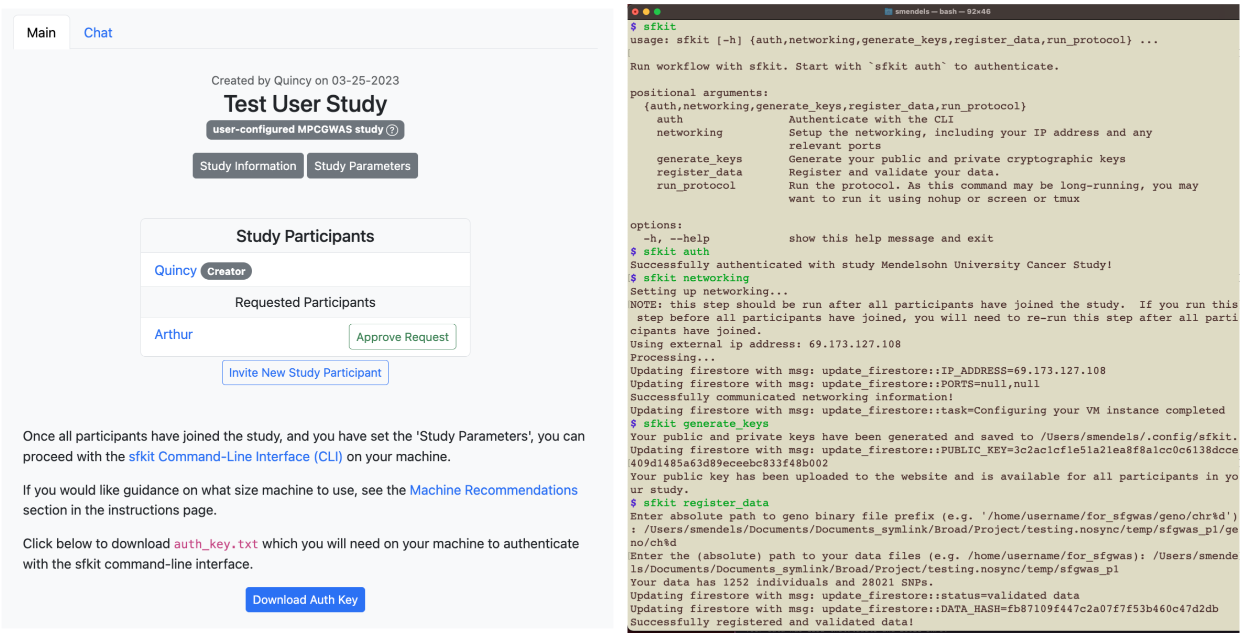

Supplementary Figure 5: **Screenshots of a *user-configured* study: study web-page (left) and command-line interface (right).** The left image illustrates a sample study utilizing the *user-configured* option. On this page, study participants would click buttons to edit parameters, add new participants, and download their authentication key. The right image displays example usage of sfkit command-line interface (CLI), including authentication, setting up networking, generating keys, and validating data. The only remaining step for this user would be executing the actual protocol (with `sfkit run_protocol`). After the study finishes, the users can view the results either on their machine or in the UI of the study page.

**Dataset:** eMERGE (31,292 samples × 38,040,168 SNPs)										
**Workflow:** SF-GWAS **Setting:** 7 co-located users **Machines:** GCP, 16CPUs, 128GB										
	Setup		Protocol execution						Total	
			Quality Control		PCA		Assoc. Test			
	Time	Comm.	Time	Comm.	Time	Comm.	Time	Comm.	Time	Comm.
	10	0.0000001	90	600	420	747	390	388	910	1735

**Dataset:** 1000 Genomes Project (2,504 samples × 28,021 SNPs)											
**Nbr. of Users:** 2 **Machines:** 16CPUs, 128GB											
Workflow	Setting	Setup		Protocol execution						Total	
				Quality Control		PCA		Assoc. Test			
		Time	Comm.	Time	Comm.	Time	Comm.	Time	Comm.	Time	Comm.
SF-GWAS	co-located	10	0.0000001	1	0.3	90	51	9	2.1	110	53.40
	non-co-loc.	10	0.0000001	1	0.3	123	51	9	2.1	143	53.40
SF-PCA	co-located	10	0.0000001	NA	NA	40	23	NA	NA	50	23.00
	non-co-loc.	10	0.0000001	NA	NA	106	23	NA	NA	116	23.00
MPC-GWAS	co-located	40	0.06	18	0.4	55	7.6	4.2	0.1	117.2	8.16

**Dataset:** Lung Cancer (9,178 samples × 612,794 SNPs)											
**Setting:** 2 co-located users **Workflow:** SF-GWAS											
Samples	CPUs, RAM	Setup		Protocol execution						Total	
				Quality Control		PCA		Assoc. Test			
		Time	Comm.	Time	Comm.	Time	Comm.	Time	Comm.	Time	Comm.
9178	16, 128GB	10	0.0000001	6	6	111	30	27	8	154	44
	32, 256GB	10	0.0000001	4	6	65	30	20	8	99	44
18356	16, 128GB	10	0.0000001	6	6	208	50	47	9.7	271	65.7
	32, 256GB	10	0.0000001	4	6	130	50	35	9.6	179	65.60
27534	16, 128GB	10	0.0000001	6	6	208	50	47	9.7	271	65.7
	32, 256GB	10	0.0000001	6	6	123	50	33	9.6	172	65.60
36712	16, 128GB	10	0.0000001	6	6	472	69	63	11.4	551	86.4
	32, 256GB	10	0.0000001	6	6	200	68	47	11.3	263	85.30

Supplementary Table 1: **Evaluation of sfkit's performance across a range of study settings.** We report sfkit's runtime (in minutes) and communication cost (in GB) for all our experiments conducted on the eMerge, 1000 Genomes Project and Lung Cancer datasets. *Setup* includes the study creation and configuration, the computational setup, and the key exchange and data validation from the study execution step (see the Usage Process section in Materials and Methods). We also provide the runtime for the main steps in the protocol execution step. The communication cost is reported for one user. For non-co-located experiments, we used the *user-configured* mode, while for the co-located experiments, we used the *auto-configured* mode. The setup took only a few minutes for all experiments, whereas the runtime for secure computations increased with the size of the participants' datasets. When using machines hosted by different providers (i.e., GCP and Azure), sfkit's runtime increased due to added communication overhead. Note that in the *user-configured* mode the users are expected to configure their own machine, which is expected to take more effort than sfkit's automated setup.

References

[1] Hyunghoon Cho, David J Wu, and Bonnie Berger. Secure Genome-Wide Association Analysis using Multiparty Computation. *Nature biotechnology*, 2018. [PubMed: 29734293] [PubMed Central: PMC5990440] [doi:10.1038/nbt.4108].

[2] Hyunghoon Cho, David Froelicher, Jeffrey Chen, Manaswitha Edupalli, Apostolos Pyrgelis, Juan R Troncoso-Pastoriza, Jean-Pierre Hubaux, and Bonnie Berger. Secure and Federated Genome-Wide Association Studies for Biobank-Scale Datasets. *bioRxiv*, pages 2022–11, 2022. [doi:10.1101/2022.11.30.518537].

[3] Jian Yang, Sang Hong Lee, Michael E Goddard, and Peter M Visscher. GCTA: a tool for genome-wide complex trait analysis. *American journal of human genetics*, 88(1):76–82, 2011. [PubMed: 21167468] [PubMed Central: PMC3014363] [doi:10.1016/j.ajhg.2010.11.011].

[4] Qing Lan, Chao A Hsiung, Keitaro Matsuo, Yun-Chul Hong, Adeline Seow, Zhaoming Wang, H Dean Hosgood, 3rd, Kexin Chen, Jiu-Cun Wang, et al. Genome-wide association analysis identifies new lung cancer susceptibility loci in never-smoking women in Asia. *Nat. Genet.*, 44(12):1330–1335, December 2012. [PubMed: 23143601] [PubMed Central: PMC4169232] [doi:10.1038/ng.2456].

[5] Shaun Purcell, Benjamin Neale, Kathe Todd-Brown, Lori Thomas, Manuel A R Ferreira, David Bender, Julian Maller, Pamela Sklar, Paul I W de Bakker, Mark J Daly, and Pak C Sham. PLINK: a toolset for whole-genome association and population-based linkage analysis. *American Journal of Human Genetics*, 81:559–575, 2007. [PubMed: 17701901] [PubMed Central: PMC1950838] [doi:10.1086/519795].
